# Complementary Role of Fishers’ Experiential Knowledge to Conventional Science in Terms of Species-Specific Biological Traits and Population Changes in Azorean Waters

**DOI:** 10.3390/biology12020194

**Published:** 2023-01-26

**Authors:** Régis Santos, Ualerson Iran Peixoto, Morgan Casal-Ribeiro, Wendell Medeiros-Leal

**Affiliations:** 1 Institute of Marine Sciences—Okeanos, University of the Azores, Rua Prof. Dr. Frederico Machado, 4, 9901-862 Horta, Portugal; 2 Institute of Marine Research—IMAR, University of the Azores, Rua Prof. Dr. Frederico Machado, 4, 9901-862 Horta, Portugal

**Keywords:** local ecological knowledge, small-scale fisheries, historical changes, marine species, Atlantic Ocean

## Abstract

**Simple Summary:**

Combining scientific data with knowledge held by fishers may be a good way to help people make decisions about marine ecosystem conservation. The goal of this study was to assess how much fishers in the Azores know about the biology and ecology of six commercially important marine species. This knowledge was then compared to the scientific information already out there to understand its value and complementarity. In the nine islands that make up the archipelago, 105 fishers were interviewed. Findings revealed a reasonable level of agreement between the information provided by fishers and the scientific literature. This reinforced how useful fishers’ experiential knowledge is, especially when scientific information is limited. However, more research should be conducted to ensure the results are more reliable and consistent.

**Abstract:**

Combining scientific information with fishers’ perceptions may be a robust approach for directing decision-makers working with marine ecosystems. This is particularly the case when baseline data on a vulnerable stock are poor, as the integration of fishers’ experiential knowledge can help fill data gaps, as well as inform legitimate management actions, and empower fishing communities in resource management. This study aimed to analyze fishers’ knowledge regarding the biology (reproduction, growth, and maximum size) and temporal changes in the abundance and size of six commercially important marine species (red porgy *Pagrus pagrus*, veined squid *Loligo forbesii*, blue jack mackerel *Trachurus picturatus*, blackspot seabream *Pagellus bogaraveo*, blackbelly rosefish *Helicolenus dactylopterus*, and European conger *Conger conger*) in the Azores small-scale communities. Additionally, a comparison between fishers’ knowledge and available scientific information was performed to determine the former’s value and its possible complementarity with the latter. A total of 105 fishers were surveyed in the nine islands of the archipelago. The results demonstrated a reasonable level of agreement between the information from fishers and scientific literature on the species-specific spawning seasons and growth rates. The median values of size at maturity and maximum length were not statistically different between data sources. Most participants indicated size and abundance trends that were consistent with the literature. This study highlights the usefulness of fishers’ perceptions in improving knowledge about species characteristics and temporal changes in commercially exploited stocks, especially when scientific research is limited, but further research should be encouraged to improve the reliability and consistency of these results.

## 1. Introduction

Small-scale fisheries (SSF) are traditional fisheries involving fishing families (as opposed to commercial companies) and relatively small boats (if any), which perform short fishing trips close to the coast and catch fish primarily for local consumption [1]. In addition to having social and cultural significance, these fisheries globally provide livelihood and nutritional security for millions of small-scale fishers and local communities [2,3]. Despite this, SSF are often understudied, which implies a severe constraint not only on assessing the magnitude of their impacts on exploited resources but also on effective suggestions made for their sustainable exploration [4,5,6]. To add to the problem, small, unassessed fisheries have been estimated to be in substantially worse condition than assessed fisheries [7].

In Portugal, coastal areas that have historically been centered on small-scale fishing communities–such as those in the Azores–have historically relied on fishing as a major source of income. The Azores is a remote Portuguese archipelago composed of nine volcanic islands located in the northeast Atlantic Ocean, where fishing activity employs over 3000 people. Since 2010, the region has had average landings of 10 M tons year^−1^ and a value of around 34 M €, which represents around 13% of the total value of fisheries in Portugal [8]. The fishery developed in the Azores is multi-species and multi-gear and has its dynamics apparently driven by the main target species, such as blackspot seabream *Pagellus bogaraveo*, tuna, and tuna-like species. Other important species include blackbelly rosefish *Helicolenus dactylopterus*, veined squid *Loligo forbesii*, European conger *Conger conger*, alfonsinos *Beryx* spp., blue jack mackerel *Trachurus picturatus*, and red porgy *Pagrus pagrus* [9]. However, the sustainability of current harvest levels on a significant portion of the exploited stocks remains unassessed under the European Union (EU) Marine Strategy Framework Directive (MSFD) and the United Nations (UN) Sustainable Development Goals (SDG) [10]. This is in large part due to the lack of sufficient reliable scientific data on these resources. Gathering such data is also particularly challenging, given the large number of fishing vessels that employ a variety of fishing gear and catch a wide range of species [9].

When there is insufficient baseline data on a fishery and its exploited species for an analytical assessment, scientists often base their recommendations for management on the precautionary principle. According to EU law, the precautionary approach “means an approach in which the absence of adequate scientific information should not justify delaying or failing to take management measures to conserve target species, associated or dependent species, non-target species, and the environment” [11]. This comes from the UN Fish Stocks Agreement, to which the EU is a contracting party [12], and its adoption may imply a reduction in fishing opportunities when the biological reference points or proxies are unknown. In the Azores, this occurred, e.g., with *T. picturatus* and *Beryx* spp. fisheries, for which the International Council for the Exploration of the Sea (ICES) considers that a “precautionary reduction of catches should be implemented unless there is ancillary information clearly indicating that the current level of exploitation is appropriate for the stock” [13,14].

The comprehensive and location-specific expertise possessed by many fishers presents an opportunity to produce baseline knowledge in these data-deficient situations and improve assessment and conservation initiatives [15,16,17]. In this context, fishers’ experiential knowledge (FEXK) has been widely used to understand multiple aspects associated with fishing dynamics, fish behavior, reproduction, as well as temporal changes in the abundance and size of fishery resources (e.g., [18,19,20,21,22,23,24]). Such collaborations between scientists and fishers improve transparency and communication, resulting in increased confidence and legitimacy among fishers in science-based management measures [15,25,26,27]. However, FEXK should not be accepted without examination, and its limits should be thoroughly explored by well-designed research [28].

This study’s objective was to analyze FEXK concerning the biology (reproductive aspects, growth, and maximum size) and temporal changes in the abundance and length distributions of six commercially important marine species (*P. pagrus*, *L. forbesii*, *T. picturatus*, *P. bogaraveo*, *H. dactylopterus*, and *C. conger*) in the Azores. In addition, a comparison between FEXK and the available scientific information was performed to determine the former’s value and possible complementarity with the latter. This is the first study of its kind to be conducted in the Azores area and its findings may represent an important milestone in integrating research on fishers’ knowledge into regional science and management.

## 2. Material and Methods

### 2.1. Data Collection

Fishers from the nine islands of the Azores were surveyed between January and May 2022. Questionnaires were performed with the help of the administrative staff and presidents of fishers’ associations, with fishers being selected through purposive sampling based on their availability to answer the questionnaire. All participants were informed of the study objectives and the intended use of the collected data. To safeguard confidentiality, questionnaires were kept anonymous.

Questionnaire items related to general information about fisher’s experience and age, as well as the biological characteristics of the six studied species, such as size at maturity (at what size does this species start reproducing?), reproductive season (at what time of the year–spring, summer, autumn or winter–does this species reproduce?), maximum size (what the size of the biggest individual of this species ever caught?), and whether the species grows slowly, moderately, or rapidly. Items also covered fishery-related aspects, such as changes in length and abundance (catch rate) since the fisher started fishing (since you started fishing, have sizes/catch rates of this species increased, decreased, or remained stable?).

### 2.2. Data Analysis

Data reported in weight by fishers were initially transformed into lengths by applying weight–length equations [29,30]. Differences between fishers’ reports and scientific literature regarding the size at maturity and maximum size were verified by comparing the median values through the Mann–Whitney U test (Wilcoxon Rank Sum test, *W*), using the *stats* package in R [31]. The significance level was set at a *p*-value < 0.05. The information on species-specific spawning season, growth rate, and trends in the size and abundance estimates–derived from scientific surveys and fishery-dependent standardized catch-per-unit-effort were compared between the two data sources by using four arbitrary measures of agreement previously described by Ribeiro et al. [21]: high agreement (HA), when the information provided by more than 70% of the surveyed fishers was in agreement with the literature; medium agreement (MA), when this rate was between 30% and 69%; low agreement (LA), when it was less than 29%; and no agreement (NA), if none of the interviewed fishers provided information in agreement with the scientific literature.

Only data from fishers who answered at least one question were included in the analyses. Hence, the number of fishers surveyed may vary between the analyzed items and studied species.

## 3. Results

**Figure 1:**
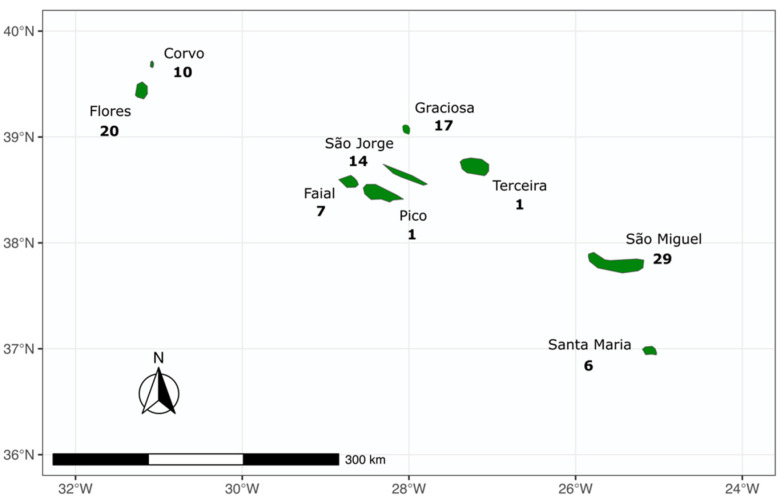
Spatial coverage of the sampling procedure performed in the Azores archipelago involving fishers. The numbers indicate the absolute sample size (*n*).

**Figure 2:**
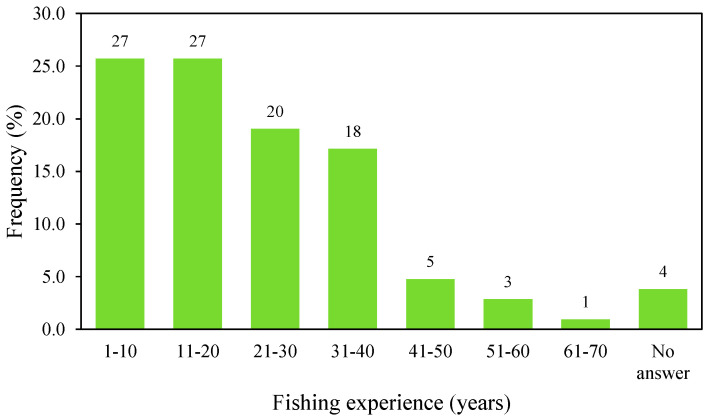
Fishing experience of the surveyed Azorean fishers in this study. The numbers above the columns indicate the absolute sample size (*n*).

A total of 105 fishers were surveyed from the nine islands of the Azores: 29 from São Miguel, 20 from Flores, 17 from Graciosa, 14 from São Jorge, 10 from Corvo, seven from Faial, six from Santa Maria, one from Pico, and one from Terceira (Figure 1). Participants were on average 47 years of age (s.d. = 10.3 years) and showed relevant fishing experience, with 45% of them starting their fishing activity more than 20 years ago (Figure 2).

The information on the spawning season from fishers’ knowledge and scientific literature showed a high level of agreement for four species (*L. forbesii*, *P. bogaraveo*, *H. dactylopterus*, and *C. conger*), while the other two species showed a medium level (*P. pagrus* and *T. picturatus*) (Table 1). Since the scientific literature does not report a single season for any of the species but rather a range, e.g., winter–spring (Table 1), this comparison considered aggregate fishers’ responses, i.e., winter plus spring, as an alternative approach for this analysis.

**Figure 3:**
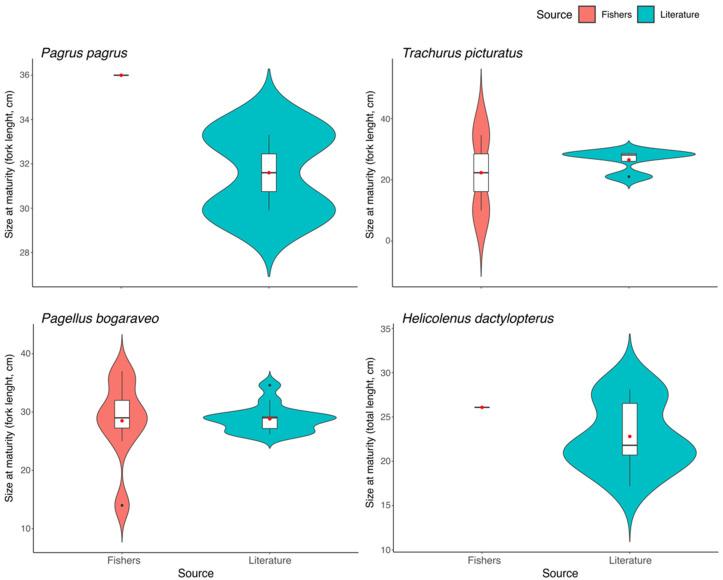
Size at maturity (cm) of the species studied in the Azores according to fishers’ experiential knowledge and obtained from scientific literature [32]. Line inside the box represents the median, the box represents the interquartile range, the curves indicate the kernel probability density of the data at different values, and the red point represents the mean value. *P. pagrus* (no. of fishers’ responses = 1), *T. picturatus* (*n* = 2), *P. bogaraveo* (*n* = 8), *H. dactylopterus* (*n* = 1).

Most surveyed fishers did not answer the item regarding size at maturity. Only one response was obtained for *P. pagrus* and *H. dactylopterus*, two for *T. picturatus*, and eight for *P. bogaraveo*. Considering the comparison between the median sizes reported for these species by the fishers and existing literature, no significant differences were observed between them (*P. pagrus*: *W* = 2.0, *p* = 0.540; *T. picturatus*: *W* = 4.0, *p* = 1.000; *P. bogaraveo*: *W* = 62.5, *p* = 0.681; *H. dactylopterus*: *W* = 9.0, *p* = 0.499; Figure 3). No information on length associated with the spawning size of *L. forbesii* and *C. conger* was provided by the fishers, nor was it available in the literature.

**Figure 4:**
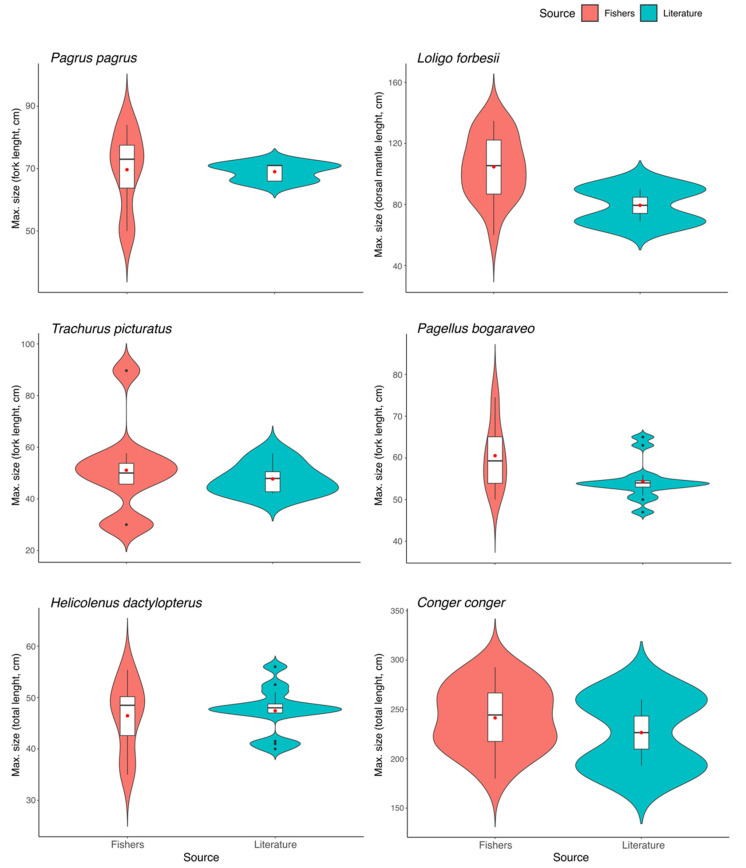
Maximum size (cm) of the species studied in the Azores according to fishers’ experiential knowledge and obtained from scientific literature [32]. Line inside the box represents the median, the box represents the interquartile range, the curves indicate the kernel probability density of the data at different values, and the red point represents the mean value. *P. pagrus* (no. of fishers’ responses = 14), *L. forbesii* (*n* = 20), *T. picturatus* (*n* = 9), *P. bogaraveo* (*n* = 13), *H. dactylopterus* (*n* = 8), *C. conger* (*n* = 19).

Considering the maximum sizes reported for the six species, no significant differences were observed between the median values from the different data sources (*P. pagrus*: *W* = 44.0, *p* = 0.428; *L. forbesii*: *W* = 33.0, *p* = 0.152; *T. picturatus*: *W* = 39.0, *p* = 0.458; *P. bogaraveo*: *W* = 137.5, *p* = 0.067; *H. dactylopterus*: *W* = 60.0, *p* = 0.810; *C. conger*: *W* = 24.0, *p* = 0.590; Figure 4).

Fishers’ knowledge on growth rates showed a medium level of agreement with the literature for *P. bogaraveo* and *H. dactylopterus*, while *P. pagrus* and *T. picturatus* showed a low level of agreement (Table 2). No scientific information on the growth rate of *L. forbesii* and *C. conger* was available, and FEXK constituted the only source of information.

Most participants indicated a stable trend in the size of the six species between the present and when they started fishing (Table 3). This was consistent with the scientific literature available for *P. bogaraveo*, *H. dactylopterus*, and *C. conger*, being demonstrated by a medium level of agreement for the first two and a high level for the last (Table 3). No scientific information on size trends was available for the other studied species. Catch rates of *L. forbesii*, *T. picturatus*, and *C. conger* remained stable according to most surveyed fishers, whereas for *P. pagrus*, *P. bogaraveo*, and *H. dactylopterus* this trend is declining (Table 4). These data agreed with the literature available for *P. bogaraveo*, *H. dactylopterus*, and *C. conger*, for which the level of agreement was considered moderate (Table 4). For *P. pagrus*, the scientific literature has shown an increasing pattern in the catch rate, while for *T. picturatus*, this pattern is decreasing (Table 4). The level of agreement noted between the literature and fishers’ knowledge for these species was low and medium, respectively. For *L. forbesii*, FEXK was the only source of information available on catch trends (Table 4).

## 4. Discussion

This study showcases the first results of an evaluation of the complementary role of FEXK to conventional science in the Azores. The results provided evidence that fishers’ understandings constitute important resources for describing biological traits, as well as long-term changes in the abundance and length of target species (such as *P. pagrus, L. forbesii, T. picturatus, P. bogaraveo, H. dactylopterus*, and *C. conger*). Overall, there was some level of agreement between FEXK and the scientific literature. Such a convergence encourages the coupling of these different sources of knowledge in the context of management developments.

Through FEXK, it was possible to identify some reproductive characteristics of studied species. Local fishers often acquire their knowledge about these attributes by catching juveniles and observing the physical condition and appearance of mature individuals during the catching process [41]. From these observations, fishers may be able to identify growth and reproduction areas as well as maturity and spawning seasons [18]. Nevertheless, several studies have shown inconsistencies or reduced agreement between FEXK and scientific literature on fish reproduction [21,42,43]. This problem may be partially due to the short spawning period of the caught species, or to the fact that fishers are mostly collecting juveniles (immature fish) owing to a drop in the number of adult fish [42].

Despite the reasonable agreement between FEXK and scientific literature observed in the present study, most participants did not respond to questions about size at maturity and spawning season. This may be related to the way fish are predominantly commercialized in the Azores, i.e., whole with viscera, preventing a more precise identification of the reproductive condition of the species. Therefore, there is a need for further detailed studies regarding reproduction–preferably using both FEXK and biological knowledge. This is especially important for species, such as *L. forbesii* and *C. conger*, about which we still know very little concerning these aspects. The collaborative and participatory monitoring of species reproduction and engaging fishers in data collection might be a good approach for conducting such studies [15,21,44].

The maximum length estimates were comparable to those documented in scientific literature for the six investigated species. These results, in conjunction with those from reproduction, corroborate previous studies demonstrating that FEXK can provide valuable data on strategic life history parameters, especially for species that are important to fishers as food and income sources [20,21,45,46]. In addition to their large sizes, most species studied here are characterized in the literature by their slow growth [32,47]. Growth rate is the intrinsic rate of population growth that should occur in a population under natural circumstances (i.e., no fishing) [33,48]. According to Hutchings [49], the ability of a fish population to sustain fishing mortality and recover after its collapse is ultimately determined by the population growth rate. Because of this, the population growth rate can be used to assess the intrinsic vulnerability and extinction risk of fish species [50,51] and has been adopted as one of the most relevant indicators by the International Union for Conservation of Nature Red List of Threatened Species (IUCN Red List), as well as many national guidelines and criteria (e.g., American Fisheries Society) [52]. However, in practice, population growth rate is very difficult to estimate, especially, for species without demographic data [51,53]. In the present study, fishers may also have reflected this difficulty in some way, which ultimately resulted in a medium to low level of agreement with the scientific literature. Some possible solutions include using relationships between the growth rate and life-history traits [54] that are easier for fishers to identify and report (e.g., maximum length, lifespan).

Besides identifying and recognizing life-cycle traits of species, effective fisheries management and stock recovery are linked to the perception of changes in the number and size of individuals in an exploited population. In this context, FEXK may be valuable in identifying stocks that require further data collection, as it can help to identify and prioritize species in which a decline in abundance or size has been reported by fishers. Fishers who were surveyed in this study reported a general decline in abundance (catch rates) of some species, such as *P. bogaraveo* and *H. dactylopterus*, since these fishers first started fishing. In fact, these species’ populations have been acutely diminishing in recent years [35,39,40,55], and a variety of fishing-control measures, such as total allowable catches/quotas and minimum landing sizes, have been put in place in the Azores [32,39]. The temporal stability in size (length) for these species described in the literature [35,39] was also reflected in information provided by participants, suggesting that those management measures would be somewhat efficient in maintaining the populations’ size structure. However, the situation may become worse when FEXK is the only information available, as it is in the cases of *L. forbesii* for abundance and length, and *P. pagrus* and *T. picturatus* for lengths. Fishers’ current assessment of these species’ status is one of stability; no temporal variations have been detected in the variables investigated. On the other hand, as most studied species are highly vulnerable to disturbance because of their late maturity, long lifespan, low fecundity, and slow growth [32], and due to the importance of these species to the Azores in terms of landed value and weight [9], it would be desirable to perform more studies and continuously monitor the population status.

The results of this study reinforce the potential applications of FEXK as a complementary (and often unique) data source in SSF, such as the Azores [24]. However, several obstacles still constrain the adoption of FEXK into fisheries management, especially concerning collecting data from fishers. Fishers from different generations may have different perspectives on resource conservation status and may use biased information to judge changes in the environment (shifting baseline syndrome; [56]). Studies have documented this phenomenon in SSF [57], although some fishing communities may have attenuated potential patterns related to shifting baselines [19,21]. Low statistical representativity prevented assessing this effect in the Azores during the present study, but future research should strive to do so.

Improving fisher participation in research initiatives is crucial, as it currently seems to be undermined by strained relationships and a lack of trust in the scientific/management communities [58]. Measures should be taken to ensure the quality of FEXK data collected, by adopting the best available marine social sciences practices [59], using well-planned participation as a research tool [60], and by avoiding research fatigue that causes fishers to become reluctant in involving themselves in research [61]. If successful, ongoing efforts to integrate FEXK in the Azores (among other research involving fishers) should advance knowledge about regional stocks and improve their management, build positive relationships between fisheries stakeholders, and put in place management decisions that are more widely accepted and adopted [62].

## 5. Conclusions

This study showed that obtaining information on the biology and temporal variations in the abundance and size of commercially important marine species in the Azores using FEXK interviews may be a timely, efficient, reliable, and cost-effective method when scientific data are not available. This conclusion was based on a cross-analysis of fishers’ knowledge with scientific literature, which reinforced the established worldwide applications of FEXK to fisheries and ecological sciences. Participants reported reproductive characteristics, such as size at maturity and spawning season, as well as temporal changes affecting size, as being the sole source of information for certain studied species in the Azores. These results had limitations, particularly in terms of the number of surveyed fishers, but nevertheless represent an important baseline for future studies in the region. Despite the challenges of implementing collaborative efforts with local stakeholders and integrating FEXK into biological research and management, it is essential to underscore that FEXK-based studies may improve local capacity, refine stock assessment initiatives, and increase the likelihood of success for future conservation actions, particularly in small-scale communities. Further research on the biological and ecological traits and FEXK of the species targeted by the Azorean fisheries should be encouraged to improve the reliability and consistency of results, including a larger sample size and temporal scale.

## Figures and Tables

**Table 1:** Spawning season of the six species studied in the Azores according to fishers’ experiential knowledge, obtained from scientific literature [32], and the agreement between these two sources of information. MA = medium agreement; HA = high agreement. Nº responses = number of fishers who answered this item.

Species	Spawning Season (Fishers)	Nº Responses	Frequency (%) *	Spawning Season (Literature)	Agreement
*Pagrus pagrus*	Spring	4	23.5	Spring to summer	MA
	Summer	3	17.6		
	Autumn	0	0.0		
	Winter	10	58.8		
*Loligo forbesii*	Spring	2	13.3	Year-round, peak between the Winter and Spring	HA
	Summer	1	6.7		
	Autumn	0	0.0		
	Winter	12	80.0		
*Trachurus picturatus*	Spring	3	18.8	Winter to spring	MA
	Summer	9	56.3		
	Autumn	0	0.0		
	Winter	4	25.0		
*Pagellus bogaraveo*	Spring	4	23.5	Winter to spring	HA
	Summer	1	5.9		
	Autumn	0	0.0		
	Winter	12	70.6		
*Helicolenus dactylopterus*	Spring	4	23.5	Winter to summer	HA
	Summer	5	29.4		
	Autumn	0	0.0		
	Winter	8	47.1		
*Conger conger*	Spring	3	23.1	Winter to summer	HA
	Summer	1	7.7		
	Autumn	0	0.0		
	Winter	9	69.2		

* Considering only answered questions.

**Table 2:** Growth rate of the six species studied in the Azores according to fishers’ experiential knowledge, obtained from scientific literature [32], and the agreement between these two sources of information. k = von Bertalanffy growth coefficient, expressing the rate (year^−1^) at which the asymptotic length (i.e., the length that the individual would reach if it grew indefinitely) is approached; LA = low agreement; MA = medium agreement. Nº responses = number of fishers who answered this item.

Species	Growth Rate (Fishers)	Nº Responses	Frequency (%) *	Growth Rate (Literature) **	Agreement
*Pagrus pagrus*	Slow	5	15.6	0.04 ≤ k ≤ 0.07	LA
	Moderate	25	78.1	= Slow	
	Fast	2	6.3		
*Loligo forbesii*	Slow	2	4.8	No information	-
	Moderate	25	59.5		
	Fast	15	35.7		
*Trachurus picturatus*	Slow	3	7.3	0.07 ≤ k ≤ 0.20	LA
	Moderate	19	46.3	= Slow	
	Fast	19	46.3		
*Pagellus bogaraveo*	Slow	16	38.1	0.06 ≤ k ≤ 0.17	MA
	Moderate	21	50.0	= Slow	
	Fast	5	11.9		
*Helicolenus dactylopterus*	Slow	12	36.4	0.05 ≤ k ≤ 0.18	MA
	Moderate	19	57.6	= Slow	
	Fast	2	6.1		
*Conger conger*	Slow	4	11.4	No information	-
	Moderate	25	71.4		
	Fast	6	17.1		

* Considering only answered questions. ** k = 1.0 year^−1^ indicates fast growth, k = 0.5 year^−1^ moderate growth and k = 0.2 year^−1^ indicates slow growth [33].

**Table 3:** Size trend of the six species studied in the Azores according to fishers’ experiential knowledge, obtained from scientific literature, and the agreement between these two sources of information. MA = medium agreement; HA = high agreement. Nº responses = number of fishers who answered this item.

Species	Size Trend (Fishers)	Nº Responses	Frequency (%) *	Size Trend (Literature)	Reference	Agreement
*Pagrus pagrus*	Increasing	1	1.6	No information	-	-
	Stable	40	65.6			
	Decreasing	20	32.8			
*Loligo forbesii*	Increasing	5	7.8	No information	-	-
	Stable	52	81.3			
	Decreasing	7	10.9			
*Trachurus picturatus*	Increasing	0	0.0	No information	-	-
	Stable	50	84.7			
	Decreasing	9	15.3			
*Pagellus bogaraveo*	Increasing	4	7.4	Stable	[34]	MA
	Stable	27	50.0			
	Decreasing	23	42.6			
*Helicolenus dactylopterus*	Increasing	3	4.8	Stable	[35]	MA
	Stable	43	69.4			
	Decreasing	16	25.8			
*Conger conger*	Increasing	5	7.9	Stable	[36]	HA
	Stable	44	69.8			
	Decreasing	14	22.2			

* Considering only answered questions.

**Table 4:** Catch trend of the six species studied in the Azores according to fishers’ experiential knowledge, obtained from scientific literature (reported abundance index estimates), and the agreement between these two sources of information. LA = low agreement; MA = medium agreement. Nº responses = number of fishers who answered this item.

Species	Catch Trend (Fishers)	Nº Responses	Frequency (%) *	Catch Trend (Literature)	Reference	Agreement
*Pagrus pagrus*	Increasing	4	6.9	Increasing	[37]	LA
	Stable	24	41.4			
	Decreasing	30	51.7			
*Loligo forbesii*	Increasing	5	7.5	No information		
	Stable	39	58.2			
	Decreasing	23	34.3			
*Trachurus picturatus*	Increasing	2	3.3	Decreasing	[38]	MA
	Stable	31	51.7			
	Decreasing	27	45.0			
*Pagellus bogaraveo*	Increasing	5	8.3	Decreasing	[39]	MA
	Stable	15	25.0			
	Decreasing	40	66.7			
*Helicolenus dactylopterus*	Increasing	4	6.7	Decreasing	[40]	MA
	Stable	25	41.7			
	Decreasing	31	51.7			
*Conger conger*	Increasing	3	5.1	Stable	[36]	MA
	Stable	32	54.2			
	Decreasing	24	40.7			

* Considering only answered questions.

## Data Availability

The data underlying this article will be shared upon reasonable request to the corresponding author.
